# Chronic Traumatic Encephalopathy: Update on Current Clinical Diagnosis and Management

**DOI:** 10.3390/biomedicines9040415

**Published:** 2021-04-12

**Authors:** Kevin Pierre, Kyle Dyson, Abeer Dagra, Eric Williams, Ken Porche, Brandon Lucke-Wold

**Affiliations:** 1 College of Medicine, University of Florida, Gainesville, FL 32611, USA; kpierre150@ufl.edu (K.P.); kdyson@ufl.edu (K.D.); abeer.dagra@ufl.edu (A.D.); williamseric@ufl.edu (E.W.); 2 Department of Neurosurgery, University of Florida, Gainesville, FL 32608, USA; ken.porche@neurosurgery.ufl.edu

**Keywords:** chronic traumatic encephalopathy, new innovations, biomarkers, emerging imaging

## Abstract

Chronic traumatic encephalopathy is a disease afflicting individuals exposed to repetitive neurotrauma. Unfortunately, diagnosis is made by postmortem pathologic analysis, and treatment options are primarily symptomatic. In this clinical update, we review clinical and pathologic diagnostic criteria and recommended symptomatic treatments. We also review animal models and recent discoveries from pre-clinical studies. Furthermore, we highlight the recent advances in diagnosis using diffusor tensor imaging, functional magnetic resonance imaging, positron emission tomography, and the fluid biomarkers t-tau, sTREM2, CCL11, NFL, and GFAP. We also provide an update on emerging pharmaceutical treatments, including immunotherapies and those that target tau acetylation, tau phosphorylation, and inflammation. Lastly, we highlight the current literature gaps and guide future directions to further improve clinical diagnosis and management of patients suffering from this condition.

## 1. Introduction

Chronic traumatic encephalopathy (CTE) is a neurodegenerative disease exhibiting a distinct pattern of neuropathological changes associated with repetitive head trauma leading to increased risk of long-term memory and cognition issues [1,2,3,4,5,6,7,8,9,10,11,12,13,14,15,16]. This association between repetitive concussive and asymptomatic sub-concussive trauma and early dementia has been recognized for over 90 years now, mostly in context of routine participation in contact sports and military combat, with some reports indicating this observed pathology in people exposed to physical abuse, head-banging, and poorly controlled epilepsy [1,6,8,9,17,18]. Repetitive head trauma causes lesions at traumatic stress points leading to axonal injury, micro-hemorrhages and subsequent loss of blood brain barrier integrity. These insults trigger an inflammatory cascade and a deposition of phosphorylated tau (p-tau) protein, amyloid-beta (Aβ), TDP-43, neurofibrillary triangles, neutrophil neurites, astrocytic tangles, neuronal loss and cerebral atrophy with white matter changes. The cis isoform of the pathogenic p-tau variants catalyzes transformation of normal into pathogenic tau in a process referred to as cistauosis [1,5,6,8,9,10,11,12,13,14,15]. Clinically, CTE presents with a spectrum of symptoms including changes in behavior, cognition and motor symptoms like impulsivity, paranoia, rage behaviors, headaches, memory deficits, impaired attention, dysphagia, dysarthria, and coordination problems [1,2,3,4,5,6,7,9,10,11,12,13,14,17,18]. The evidence shows that CTE begins focally in cerebral cortex and over the decades, slowly progresses to involve widespread regions of medial temporal lobe, basal ganglia, diencephalon, and brainstem. Progression of CTE has been described in four pathological stages. Stage 1 is characterized by isolated perivascular centers of p-tau and neurofibrillary tangle depositions primarily in the sulcal depths. Stage 2 presents with more frequent pathologic patchy deposition at the depths of the sulci and neurofibrillary tangles primarily dispersed throughout superficial cortical layers. These depositions continue spreading in the following stages. However, the hallmark of stages 3 and 4 is a significantly decreasing brain weight with increasing severity of cognitive abnormalities. Stage 3 is characterized by the initial brain weight reduction due to thinning of the corpus callosum, atrophy of the cerebral cortex, mammillary bodies, thalamus, and hypothalamus, with depigmentation of the locus coeruleus and substantia nigra. Finally, in stage 4, there is significant reduction in brain weight due to widespread cerebral cortical atrophy of the medial temporal lobe, thalamus, hypothalamus, and mammillary bodies, along with complete depigmentation of substantia nigra [1,5,8,11,12,13,14,15].

Although neuropathological clinical manifestations and some histological analysis overlaps between CTE and other neurodegenerative diseases, specifically Alzheimer’s disease (AD), the emerging evidence from autopsy studies have shown distinct features that distinguish CTE from other tauopathies like AD specifically in terms of the pattern of neurofibrillary tangles and other proteins [1,5,8,12,13]. For example, Beta-amyloid (Aβ) plaques are found in 52% of individuals with CTE, however when they occur, they are typically less dense and more diffuse than in Alzheimer’s disease [5,13]. While tau protein deposition occurs primarily in the superficial cortical layers with perivascular predilection in CTE, the Alzheimer’s disease is characterized by predominant neurofibrillary tangles in deep cortical laminae [7,16].

Genetic and psychosocial risk factors associated with CTE have also been investigated recently to explore the pathogenesis of CTE as a multifactorial problem. Here, repeated mild brain trauma leads to trauma-induced tau hyperphosphorylation followed by activation of a various potential second-risk determinants like involvement of apolipoprotein E (*ApoE*) e4 allele and loss of TDP-43 nuclear function. These changes can be associated with inhibition of neuronal growth following brain injury [5,6,9,11,12,15,16]. Moreover, the progression of this neurodegenerative pathology is also influenced by the age at which an individual begins playing contact sports, and even exposure to performance-enhancing drugs [5,11,16]. Another hypothesis suggests that while initial tau deposition happens in a trauma-dependent mechanism, the continued spread of depositions possibly occurs via trauma-independent mechanisms possibly associated with increased level of inflammation, neuronal tau secretion, damaged tau clearance in extracellular cerebrospinal fluid and leakage of the BBB [1,5,8,11,14,16,19].

Like most neurodegenerative diseases, CTE can only be diagnosed by postmortem neuropathologic examination of the brain tissue revealing pathognomonic lesions consisting of irregular patterns of perivascular p-tau aggregates in neurons, astrocytes, and cell processes at the depths of the cortical sulci [1,5,8,12,13,14]. Recent studies have shown increased inflammatory involvement in CTE, specifically CD68 immunoreactive microglia and increased levels of the cytokine CCL11 in the frontal cortex compared to controls or subjects with AD [1,5,20]. These findings along with attempts to find biomarkers unique to CTE on neuroimaging offer implications for development of in-vivo methods of clinical diagnosis of CTE, hence opening doors for opportunities for early identification and progression management that improves overall outcomes.

## 2. Innovations in Diagnosis

The diagnostic criteria and neuropathologic features of CTE are well defined but perfecting in-vivo diagnosis of CTE remains elusive; CTE is only definitively diagnosed at autopsy. Studies into neurobehavioral commonalities, neuropathology, cognitive and functional symptomatology, imaging findings, and biomarkers illuminate our perspective on in-vivo diagnosis and facilitate improved characterization of the spectrum of CTE. These diagnostic innovations are summarized in Table 1.

### 2.1. Clinical Diagnostic Criteria

The core diagnostic features present in more than 70% of confirmed CTE cases without comorbidities falls into the three domains: cognitive, behavioral, mood [21,22]. Cognitive symptoms include impairments in memory and executive functioning. Behavioral symptoms include verbal and physical violent behavior, explosivity, and impulsivity. Mood symptoms often include depression. Differentiation of these various manifestations of CTE from other forms of TBI like post-concussive syndrome, remains difficult, and it is unknown whether they share neuropathological features [21]. Although clinical features can be characterized in-vivo, definitive diagnosis of CTE still requires an autopsy [23], prompting Montinegro et al. to propose a new diagnostic criterion, known as trauma encephalopathy syndrome (TES). TES as a diagnostic criterion is based on the clinical features commonly found in CTE and consist of five criteria: history of multiple impacts, absence of comorbid disease that could account for symptoms, presence of symptoms for at least 12 months, presence of at least on core clinical features (e.g., Mood, cognitive or behavioral impairment), and the presence of supportive features like decline over at least 12 months, headache, or impulsivity [22]. Still, there remains various proposed clinical classifications and varying diagnostic criteria; consensus within the academic community has not been reached [24].

### 2.2. Diagnostic Imaging

While CTE remains a histologic diagnosis, identification of radiographic signs and correlates of disease severity remains a high priority. MRI is the imaging modality of choice for evaluating structural changes in chronic TBI and CTE due to superior sensitivity compared to computed tomography (CT) and the ability to detect diffuse axonal injury. Unfortunately, recurrent structural findings, such as grey matter atrophy, ventricular enlargement, and cavum septum pellucidum, are not specific to CTE [25] and structural findings are therefore not sufficient to diagnose CTE.

Use of diffusion tensor imaging (DTI) to assess white matter integrity has become a popular tool for assisting in the diagnosis of CTE [26]. Research indicates it may be useful in determining the relationship between cognitive deficits and TBI and in distinguishing the spectrum of brain injury, even when the injury was sustained years prior [27]. Post-mortem analysis has confirmed the relationship between axonal damage and decreased fractional anisotropy (FA) [28].

Blood oxygenation level-dependent MRI, also known as functional (fMRI) can detect changes in the oxygenation of hemoglobin while correlating to brain function during a specific task. Although the use of BOLD fMRI for the study of TBI presents challenges due decreased cerebral blood flow found in TBI, it remains a novel tool for analyzing the activity of specific regions of the brain [27]. Significant developments are taking place with the use of PET for diagnosing CTE [12] using ligands that bind pathology in CTE. The most well-studied is FDDNP, which binds to neurofibrillary tangles. However, it is non-specific because it binds to β-amyloid in addition to hyperphosphorylated tau [29]. Other tracers include T807, AV1451, and flortaucipir. Though nascent in their development, they are emerging as promising tools within the field of imaging biomarkers for CTE. While studies investigating this imaging modality have shown varied regions of uptake, they have consistently demonstrate increased tracer uptake in the limbic system and temporal lobe. However, evidence for tracer uptake in cortical regions widely varies [29].

### 2.3. Fluid Biomarkers

The identification of fluid biomarkers from blood or CSF is under active investigation for pre-morbid diagnosis of CTE. While no single fluid biomarker is sufficient or approved for diagnosis, several studies are continually investigating further fluid biomarker candidates [1]. Although also associated with chronic neurodegenerative disease such as AD, preliminary research supports the use of plasma t-tau as a marker of injury severity following repetitive head trauma [1,30]. Total plasma and CSF tau concentrations have been shown to correlate with increased exposure to athletic head impacts in multiple studies, and a plasma concentration ≥3.56 pg/mL has been suggested as a threshold to reliably distinguish NFL players from healthy controls [30,31,32]. Stern et al. demonstrated that exosomal tau could also reliably differentiate former NFL players from healthy controls and that levels correlate with cognitive and psychomotor decline [33]. Triggering receptor expressed on myeloid cells 2 (TREM2) is a protein involved in microglia resolution of CNS inflammation and is a known correlate of AD severity [34,35]. Microglia are known mediators of neurinflammation and have been found to contribute to the accumulation of tau in CTE [36]. Examining soluble TREM2 (sTREM2) concentrations in the CSF as a marker of microglial activation, Alosco et al. found sTREM2 correlated with total tau levels, and strengthened the relationship with tau and head trauma when included in regression models. Microglia also express cognate receptors for the chemokine CCL11/eotaxin, an inflammatory marker associated with neurodegeneration. CCL11 has been shown to be significantly elevated within the dorsolateral frontal cortex (DLFC) of former American football players, with a non-significant increase within the CSF, and could reliably differentiate CTE from AD [20]. Other promising fluid biomarkers under investigation include neurofilament light chain (NFL) and glial fibrillary acidic protein (GFAP).

## 3. Innovations in Clinically Oriented Treatment

Prevention of TBI remains the only method of prevention. Within the contact sports, which are common facilitators of repetitive mild TBI (rmTBI), preventative measures include contact rule changes and protective equipment, with an acknowledgement that no protective equipment can prevent a concussion [18]. Immediate removal from play with strict supervised return to play guidelines and proper medical management remains a crucial element in prevention of second impact syndrome and other sequalae [33].

Treatment of CTE is currently mainly supportive. However, recently elucidated understandings of neurobiological mechanisms in rodent models have led to advances in treatment development. We summarize the currently recommended treatments and promising innovations in the following paragraphs and Table 2.

### 3.1. Recommended Supportive Treatments

Non-pharmaceutical management recommendations include cognitive rehabilitation, motor therapy, mood and behavior therapy, mindfulness, the Mediterranean diet, and aerobic exercise. Vestibular rehabilitative therapy is also recommended for those with inner ear injury resultant from repetitive TBI. Occupational-ocular therapy is recommended for those with visual disturbances. There are no FDA-approved medications for CTE. They are used “off-label” and primarily target symptomology. Drugs used for memory impairment parallel those used in Alzheimer’s disease like galantamine, donezepil, and rivastigmine. In addition to stimulants like methylphenidate, dopamine agonists like carbidopa/levodopa, pramipexole amantadine, memantine, may treat apathy. Furthermore, these stimulants can treat impaired attention. Depression and anxiety medications include sertraline and escitalopram, though they with caution considering its side effect of suicidality as suicide is well-documented in CTE.

Furthermore, it is recommended to optimize drug regimens to minimize drugs interactions and reduce those that may exacerbate symptoms and cause further cognitive impairments, like sedatives and anticholinergics [37].

Considering the recent advances in the understanding of the molecular and inflammatory cascades leading to progressive neurodegeneration, pre–clinical studies paving the way for clinical trials for patients with CTE [12].

### 3.2. Review of Pre-Clinical Animal Models

CTE is characterized by progressive neurodegeneration in the absence of further trauma through incompletely understood secondary injury cascades. A better understanding of these molecular pathways and optimized treatment development would be facilitated using animal models representing CTE. While several well-characterized animal models, like the non-impact head acceleration, blast wave, weight drop, fluid percussion, and controlled cortical impact (CCI) models exist for traumatic brain injury, animal model development for CTE is still in the early stages as none fully reflect the known progressive pathological, neurocognitive, and psychiatric findings. Still, many models inflicting repetitive mild TBI reflect some findings like neurofibrillary tangles, Aβ, phosphorylated tau, and TDP-43 deposition, microgliosis, astrogliosis, ER stress, glutamate excitotoxicity, and white matter changes, as well as the sequelae involving progressive cognitive impairment and mood changes [38,39,40,41,42,43,44,45,46,47,48,49,50,51,52,53].

### 3.3. Targeting Tau Acetylation

Pre-clinical studies have investigated the treatment of tauopathies by targeting known molecular pathways. P-tau accumulation results from tau phosphorylation, preceded by ac-tau post-translational acetylation, which is likely a result of neuroinflammation, endoplasmic reticulum, and oxidative stress [49,54,55]. Studies investigating the treatment of tauopathies have largely targeted this pathway. For example, salsalate reduced inflammation, promoted neuroprotection and neurogenesis through gene upregulation, prevented hippocampal atrophy, and led to functional recovery in acetylation in mTBI rodent models by reducing p300 lysine acetyltransferase, inhibiting tau acetylation on lysine 174, and inhibiting microgliosis [55,56,57]. Salsalate treatment also proved similar results in a rodent model of frontotemporal dementia [55]. Similarly, methylene blue, which modulates K280/K281 acetylation activity, promoted neuroprotection, reduced behavioral deficits and mood changes, and minimizes neuronal degeneration, neuroinflammation, lesion volume, microgliosis, and mitochondrial dysfunction in TBI rodent models [58,59,60,61,62,63,64]. In-vitro studies demonstrated that histone deacetylase 6 (HDAC) and sirtuins (SIRT1 and SIRT2) promoted tau deacetylation, potentially representing an additional treatment methodology targeting the same pathway mechanism differently [65,66].

### 3.4. Targeting Tau Phosphorylation

Kinase inhibition has also been successfully explored in pre-clinical models. Glycogen synthase 3 beta (GSK-3B) which is activated by p-tau to cause further tau phosphorylation, promote amyloid-b related cell death, and downregulate antioxidant defenses like Nuclear factor E2-related factor 2 (Nrf2) [67,68,69]. Dimethyl fumarate (DMF) modulated GSK-3B activity, induced the Nrf2 transcriptional signature, and attenuated astrogliosis and microgliosis in tauopathy mouse models [70]. Lithium, which blocks GSK magnesium binding, promotes GSK phosphorylation, and blocks receptor tyrosine kinase activity, reduced neurodegeneration, maintained the blood-brain barrier’ integrity, improved cognitive outcomes, and reduced lesion sizes in TBI mouse models [71,72,73,74,75,76,77].

Additionally, Lithium may treat many CTE features like mood, impulsivity, suicidal ideation, and depression [78]. Valproic acid, alone or administered with lithium, results in neuroprotection and improved functional outcomes [75,79]. The GSK3 inhibitor L803-mts prevented TBI-induced depression in mTBI weight drop rodent models [72]. Intravenous simvastatin which activates the receptor tyrosine kinase to block GSK3, improved cognitive outcomes, reduced neuroinflammation, neurodegeneration, and promoted neuroregeneration in rodent models [80,81,82].

Several studies demonstrated that roscovitine and its derivative CR-8, cyclin dependent kinase (CDK) inhibitors, reduced neuroinflammation and neurodegeneration while improving functional outcomes in TBI rat models [83]. A study showed that the combined use of Lithium and roscovitine had more profound reductions in cortical and blood p-tau when used in combination rather than when used alone in a mouse model of repetitive mild TBI [50].

### 3.5. Immunotherapy

Immunotherapy by the use of monoclonal antibodies has also been studied in pre-clinical studies investigating tauopathies. Notably, a recent study demonstrated that the delivery of an adeno-associated virus (AAV) vector coding for an anti-pTau antibody reduced CNS pTau levels in rodent models of repeated traumatic brain injury [84]. An in-vitro study demonstrated that several tau antibodies successfully prevented neuronal tau uptake. The antibody 6C5 prevented interneuronal spreading and progression of aggregation after cellular uptake [85]. In an effort to avoid targeting the trans isoform of p-tau that is important for normal cellular activity, antibodies developed specific to the pathogenic cis-P-tau that develops after TBI demonstrated reductions in tauopathy and improved structural and functional outcomes [86,87,88].

### 3.6. Targeting Inflammation

Studies have also targeted the complex inflammatory cascade and metabolic changes occurring in CTE. A recent study investigated the use of 4-{2-[2-(3,4-dimethoxyphenyl)-vinyl]-6-ethyl-4-oxo-5-phenyl-4*H*-pyrimidine-1-il}benzsulfamide (OCH, a pyrimidine derivative) which is proposed to preserve mitochondrial function and proper ATP synthesis following TBI. In rodent models of repetitive TBI, OCH improved ATP-generation, respiratory intensity, and cerebral blood flow while decreasing glycolysis intensity, CTE biomarker concentrations, and β-amyloid levels. It also preserved sensorimotor function [89]. Administration of the salubrinal (SAL), a stress modulator, significantly reduced ER stress, oxidative stress, pro-inflammatory cytokines, and inducible nitric oxide synthase while preventing impulsive-like behavior in rodent models of repetitive TBI [90]. Calpain-2 is proposed to contribute to neurodegeneration following TBI. The use of a selective calpain-2 inhibitor, (C2I) significantly reduced calpain-2 activation, prevented increased tau phosphorylation and TDP-43 changes, prevented astrogliosis and microgliosis, and eliminated cognitive impairment in a rodent model of repeated traumatic brain injury [91]. Several studies have shown that the ketogenic diet enhances cognitive, motor, and pathological outcomes in rodent TBI models [92]. Pre-clinical studies have also indicated that increased recovery times in rodents improve outcomes, consistent with similar studies in human populations [93,94].

Lastly, studies have investigated the role of arachidonic acid’s metabolic product 2-arachidonoylglycerol, which inhibits inflammation started by NF-kB (2-AG). Inhibition of monoacylglycerol lipase (MAGL), which metabolizes 2-AG, significantly reduced neurodegeneration, tau phosphorylation and TDP-43 aggregation, astrogliosis, and proinflammatory cytokines while improving cognitive outcomes in a repetitive mild TBI rodent model [95]. Furthermore, 2-AG improved blood-brain barrier integrity and reduced inflammatory cytokine expression when administered exogenously in a CHI rodent model [96].

## 4. Future Discoveries

CTE has gained significant media attention in recent years. Despite significant effort, methods for pre-mortem diagnosis and treatment remain elusive. While there are several proposed clinical diagnostic criteria, there is unfortunately no consensus among the scientific community on which to use. Furthermore, there is little research assessing these criteria’s diagnostic validity.

Imaging modalities are also similarly limited. While radiographic findings are consistently identified in patients with overt clinical disease, their diagnostic utility remains unclear considering small sample sizes, inconsistent use of imaging modality, little histological correlation, and confounding and unknown effects (e.g., gender, intelligence quotient, education, genetic polymorphisms). Small sample sizes are a particularly limiting factor considering the heterogeneity of CTE among individuals. Diagnostic studies vary widely in analysis methodology, making interpretation and inter-study comparison difficult. Only two studies have directly compared histologic and imaging analyses [28,97], which would support validation of imaging-based diagnostics. Many studies do not use age-matched control groups, rendering differentiation of normal-aging associated neurological changes from CTE difficult. Many studies, particularly those analyzing contact sports, do not include females, preventing the study of gender-based outcomes of chronic TBI. Therefore, future directions to improve radiographic diagnosis of patients with CTE include: validation of diagnostic criteria; adequately controlled imaging studies with increased sample sizes and greater statistical power; inclusion of female study participants; consistent imaging methodologies; consistent use of age-matched control groups; and neuropathological evaluation to directly correlate imaging and pathologic findings. Furthermore, more studies investigating additional, more specific tau ligands like flortaucapir are needed [98]. We look forward to the results of the DIAGNOSE-CTE project, which will perform longitudinal investigations in athletes to validate various diagnostic tests [99].

While many blood and CSF biomarkers are well-characterized for TBI, few biomarkers have been studied specifically for CTE [100]. Identification of reliable biomarkers may support early diagnosis, prognostication, monitoring of disease progression, evaluation of treatment response, and support linking pathological disease burden with clinical symptomology. Considering that the pathology of CTE significantly overlaps with other tauopathies, investigating validated biomarkers in the context of CTE is a reasonable next approach [101]. While still premature, next-generation imaging modalities, such as engineered macrophages used to image micro-metastases, have potential applications to any disease state involving an inflammatory state and may have applicability to neurodegenerative diseases such as CTE [102].

Currently, there are no approved drugs that improve pathological and functional outcomes in patients with CTE. Preclinical studies have largely shown promising results in the treatment of CTE. While there are ongoing clinical trials for treatments in other tauopathies like Alzheimer’s, there are very little for CTE. Furthermore, very few pre-clinical studies explore these treatments in rodent models specific for CTE, likely due to their recent advent. Therefore, in addition to the need for continued research for rodent models accurately reflecting CTE pathology, there is a need for further treatment studies using these animal models. There is an increasingly appreciated role of the immune system in traumatic brain injury. Microglia have remained the predominant focus, however, the relative involvement of peripheral immune cells recruited to CNS after traumatic injury is unknown and further investigations into this component of disease pathogenesis may reveal novel therapeutics [97]. Other topics worthy of future investigation include development of humanized monoclonal antibodies and enhancement of blood brain barrier permeability through unilateral focused and low-intensity pulsed ultrasound methods [103].

Considering a lack of treatment options, the best treatment method for CTE remains prevention. Therefore, continued research on the efficacy of protective equipment, development of enhanced protective equipment, continued enforcement and further development of sport contact roles, and improvements and enforcement of return to play protocols is needed [104].

## Figures and Tables

**Table 1:** Innovation in Diagnosis of CTE.

Methods	Relevance
**Clinical Diagnostic Criteria**	
Trauma encephalopathy syndrome (TES)	Five criteria to make clinical diagnosis
	History of multiple impacts
	Absence of comorbid disease that could account for symptoms
	Presence of symptoms for at least 12 months
	Presence of at least one core clinical feature (mood, cognitive or behavioral impairment)
	Presence of supportive features like decline over 12+ months, headache, or impulsivity
**Imaging**	
Diffusion tensor imaging (DTI)	Detection of white matter integrity
Blood oxygenation level–dependent MRI, also known as functional (fMRI)	Detection of changes in hemoglobin oxygenation with brain task function correlation
PET	Used with CTE imaging biomarkers FDDNP, T807, AV1451, and flortaucipir. Regions of uptake depend on the specific biomarker
**Fluid Biomarkers**	
t-tau	Marker of neuroinflammation, endoplasmic reticulum, and oxidative stress
sTREM2	Marker of microglial activation
CCL11 (chemokine)	Inflammatory marker associated with neurodegeneration
Neurofilament light chain (NFL)	Marker increased in axonal injury
Glial fibrillary acidic protein (GFAP)	Glial-derived biomarker

**Table 2:** Innovation in Treatment of CTE.

Specific Agents	Mechanism
**Current Treatments**	
Cognitive rehabilitation therapy	Supportive therapy
Motor therapy	
Mood and behavior therapy	
Mindfulness	
Mediterranean diet	
Aerobic exercise	
Vestibular rehabilitative therapy	
Occupational-ocular therapy	
Memory impairment medications—galantamine, donezepil, and rivastigmine	
Stimulants—methylphenidate	
Dopamine agonists—carbidopa/levodopa, pramipexole, amantadine, memantine	
Antidepressive/anxiety medications—sertraline and escitalopram	
**Potential Treatments**	
Salsalate	Targets tau acetylation
methylene blue	
histone deacetylase 6 (HDAC)	
sirtuins (SIRT1 and SIRT2)	
Dimethyl fumarate (DMF)	Targets tau phosphorylation
Lithium	
GSK3 inhibitor L803-mts	
Intravenous simvastatin	
roscovitine (and its derivative CR-8)	
anti-pTau antibody	Immunotherapy
antibody 6C5	
OCH	Targets inflammation
salubrinal	
calpain-2 inhibitor	
2-arachidonoylglycerol (2-AG)	

## Data Availability

Not applicable.
